# Upregulation of an estrogen receptor-regulated gene by first generation progestins requires both the progesterone receptor and estrogen receptor alpha

**DOI:** 10.3389/fendo.2022.959396

**Published:** 2022-09-15

**Authors:** Meghan S. Perkins, Renate Louw-du Toit, Hayley Jackson, Mishkah Simons, Donita Africander

**Affiliations:** Department of Biochemistry, Stellenbosch University, Stellenbosch, South Africa

**Keywords:** breast cancer, estrogen receptor, menopausal hormone therapy, progesterone receptor, progestins, steroid receptor crosstalk

## Abstract

Progestins, synthetic compounds designed to mimic the activity of natural progesterone (P_4_), are used globally in menopausal hormone therapy. Although the older progestins medroxyprogesterone acetate (MPA) and norethisterone (NET) have been implicated in increased breast cancer risk, little is known regarding newer progestins, and no significant risk has been associated with P_4_. Considering that breast cancer is the leading cause of mortality in women, establishing which progestins increase breast cancer incidence and elucidating the underlying mechanisms is a global priority. We showed for the first time that the newer-generation progestin drospirenone (DRSP) is the least potent progestin in terms of proliferation of the estrogen-responsive MCF-7 BUS breast cancer cell line, while NET and P_4_ have similar potencies to estradiol (E_2_), the known driver of breast cancer cell proliferation. Notably, MPA, the progestin most frequently associated with increased breast cancer risk, was significantly more potent than E_2_. While all the progestogens enhanced the anchorage-independent growth of the MCF-7 BUS cell line, MPA promoted a greater number of colonies than P_4_, NET or DRSP. None of the progestogens inhibited E_2_-induced proliferation and anchorage-independent growth. We also showed that under non-estrogenic conditions, MPA and NET, unlike P_4_ and DRSP, increased the expression of the estrogen receptor (ER) target gene, *cathepsin D, via* a mechanism requiring the co-recruitment of ERα and the progesterone receptor (PR) to the promoter region. In contrast, all progestogens promoted the association of the PR and ERα on the promoter of the PR target gene, *MYC*, thereby increasing its expression under non-estrogenic and estrogenic conditions. These results suggest that progestins differentially regulate the way the PR and ER converge to modulate the expression of PR and ER-regulated genes. Our novel findings indicating similarities and differences between P_4_ and the progestins, emphasize the importance of comparatively investigating effects of individual progestins rather than grouping them as a class. Further studies are required to underpin the clinical relevance of PR/ERα crosstalk in response to different progestins in both normal and malignant breast tissue, to either confirm or refute their suitability in combination therapy for ER-positive breast cancer.

## Introduction

Progestins are synthetic progestogens (progesterone receptor (PR) ligands), that are classified into four consecutive generations, with the newer fourth-generation progestins reported to elicit effects more similar to natural progesterone (P_4_) than progestins from earlier generations (1–4). For example, we have shown that fourth-generation progestins, like P_4_, display anti-androgenic activity, while the earlier generation progestins display androgenic activity (5, 6). These progestins are used globally in both contraception and menopausal hormone therapy (MHT) (2–4, 7).

While both progestins and estrogens used in MHT have previously been implicated in increased breast cancer incidence (8), MHT containing progestins such as first generation medroxyprogesterone acetate (MPA) (8–12) or norethisterone (NET) (8, 10, 12), or second generation levonorgestrel (LNG) (8, 12) have been associated with a higher risk than estrogen-only MHT [reviewed in (11)]. The role of progestins in breast cancer is, however, not straightforward as some studies have suggested that progestins such as norethisterone acetate (NET-A) and LNG are not linked to increased breast cancer risk, while progestins like MPA and megestrol acetate have been used for breast cancer treatment [reviewed in (13, 14)]. An added complexity is the fact that a diverse range of progestins, known to elicit effects different to each other and P_4_, are available for therapeutic use (2, 7, 15). It is thus evident that both large-scale clinical trials and more molecular studies are required to directly compare the effects of progestins on breast cancer risk.

In addition to the estrogen receptor (ER), the PR, previously considered to only be an indicator of a functional ER in breast cancer tumors (13, 16, 17), also plays an important role in breast cancer biology [reviewed in (18)]. Although the importance of the PR in breast cancer had in fact been recognized by many research groups (19–26), particularly in terms of the roles of the PR isoforms, PR-A and PR-B, its significance has only been appreciated in recent years. The role of the PR in breast cancer is quite complex, and dependent on several factors. For instance, the unliganded PR constitutively regulates a gene profile that is distinct from the profile regulated by the progestogen-activated PR [reviewed in (27)]. Moreover, the PR can form complexes with ERα (24, 25, 28–30) and for unliganded PR-B, results in increased ERα-regulated gene expression and breast cancer cell proliferation (25). Agonist activation of both the PR and ERα, however, resulted in ERα being directed to new chromatin binding sites, leading to a gene expression profile that is associated with a good prognosis in ER-positive breast cancer (28). Consistent with a positive outcome, it has also been shown that activation of the PR by P_4_ or MPA inhibited ERα-associated gene expression in breast cancer patient-derived xenografts (31). While the two former studies did not specify the contribution of the individual PR isoforms, a study by the Greene laboratory showed that the PR isoforms differentially reprogram estrogen signaling when both the ER and PR are activated, resulting in either pro- or anti-tumorigenic effects (32).

This study aimed to directly compare the effects of P_4_ and three progestins on breast cancer cell proliferation, anchorage-independent cell growth and the expression of an ER as well as a PR target gene, while also elucidating the role of the PR and ERα. Since progestins are often co-administered with estrogens in hormone therapies (33) and breast cancer tumors often have high intratumoral estrogen levels (34), we also investigated the effects of estrogen-progestin combinations on the above-mentioned responses. Underpinning these mechanisms would further our understanding of the differential effects elicited by progestins, and whether these effects are influenced by the presence of estrogen, all of which may assist in the design of hormone therapies with fewer side-effects.

## Materials and methods

### Cell culture and inducing compounds

The human MCF-7 BUS (also known as MCF-7 BOS) breast cancer cell line was received from Prof. Ana Soto (Tufts University, Boston), and authenticated by short-tandem repeat profiling (NorthGene). The cells were maintained in Dulbecco’s Modified Eagle’s Medium (DMEM) containing 4.5 g/L glucose (Sigma-Aldrich, RSA), 5% heat-inactivated (HI) fetal calf serum (FCS) (Biochrom GmbH, Germany) and 100 IU/ml penicillin and 100 μg/ml streptomycin (Sigma-Aldrich, RSA) as previously described (35). All experiments were conducted within the first 35 passages since the cell line was thawed from storage, and only mycoplasma-negative cells were used. 17β-estradiol (E_2_), P_4_, MPA, NET, drospirenone (DRSP) and fulvestrant (ICI-182,780; ICI) were obtained from Sigma-Aldrich, RSA.

### Cell viability assays

MTT (3-(4,5-dimethylthiazolyl-2)-2,5-diphenyltetrazolium bromide) cell viability assays were conducted as previously described (36) to evaluate effects of E_2_ and the progestogens on the proliferation of the MCF-7 BUS breast cancer cell line. Briefly, MCF-7 BUS cells were seeded into 96-well plates at a density of 1 × 10^4^ cells per well in phenol red-free DMEM supplemented with 5% HI-charcoal stripped (CS)-FCS and 100 IU/ml penicillin and 100 μg/ml streptomycin. The next day the cells were treated with increasing concentrations of E_2_, P_4_, MPA, NET or DRSP, or 1 nM or 100 nM of the progestogens in the absence and presence of 1 nM E_2_ for 72 hours. Thereafter, the cells were retreated with the ligands and incubated for another 48 hours. The cells were subsequently incubated with pre-warmed MTT solution at a final concentration of 1.25 mg/ml for 4 hours. The medium was removed and 200 μL dimethyl sulfoxide (DMSO) was added to each well before the absorbance was measured at 550 nm.

### Anchorage-independent growth

Soft agar assays were conducted as previously described (36). Briefly, MCF-7 BUS cells were incubated with 1 nM or 100 nM P_4_, MPA, NET or DRSP in the absence or presence of 1 nM E_2_, or 1 nM or 100 nM E_2_ only for 21 days. Thereafter, the colonies were fixed with 37% formaldehyde and stained with 0.005% crystal violet. Colonies were quantified using ImageJ software (Version 1.49).

### Small interfering RNA transfections

MCF-7 BUS cells were seeded into 10 cm^2^ dishes at a density of 2 x 10^6^ cells in phenol red-free DMEM supplemented with 5% HI-CS-FCS and 100 IU/ml penicillin and 100 μg/ml streptomycin. The next day the cells were transfected with either 10 nM non-silencing scrambled sequence control (NSC) siRNA (Qiagen, USA) or siRNA directed against the human PR isoforms (GS5241; a combination of 4 target-specific siRNAs, Qiagen, USA), or 25 nM NSC siRNA or siRNA directed against human ERα (SC-29305; a combination of 4 target-specific siRNAs, Santa Cruz, USA), using Dharmafect transfection reagent (Dharmacon, USA) as per the manufacturer’s instructions. After 24 hours, the cells were replated into 12-well plates at a density of 2 x 10^5^ cells per well. The next day, cells were treated with 100 nM E_2_, MPA or NET for 24 hours. For the quantification of mRNA expression by real-time quantitative PCR (qPCR), total RNA was harvested, and cDNA synthesized. Reduction in protein levels was confirmed by immunoblotting.

### Real-time qPCR

MCF-7 BUS cells were plated and treated with 100 nM E_2_, P_4_, MPA, NET or DRSP, or equimolar concentrations of progestogens and E_2_ for 24 hours. Total RNA was isolated using Tri-reagent (Sigma-Aldrich, RSA) and reverse transcribed using the ImProm-II™ Reverse Transcription System (Promega, USA) as per the manufacturer’s instructions. Real-time qPCR was performed using the KAPA SYBR^®^ FAST ABI Prism qPCR Kit (Roche Applied Science, RSA) according to the manufacturer’s instructions. The mRNA expression of *CTSD* (cathepsin D), *MYC* and the reference gene GAPDH (glyceraldehyde3-phosphate dehydrogenase) was measured using the primer sets described in **Table 1**. Agarose gel electrophoresis and melt curve analyses were performed to confirm the presence of a single amplicon of the correct size (data not shown). The primer efficiency of each primer set for each cell line is shown in **Table 1**, and the relative transcript levels were determined as previously described (40).

**Table 1:** Primers used for real-time qPCR.

Gene	Primer sequence	Amplicon Length	Primer Efficiency	Ref.
***CTSD***	5’-GCGAGTACATGATCCCCTGT-3’ (fwd) 5’-CTCTGGGGACAGCTTGTAGC-3’ (rev)	89 bp	1.93	(37)
***MYC***	5’-GACGCGGGGAGGCTATTCTG-3’ (fwd) 5’-GACTCGTAGAAATACGGCTGCACCGAGTC-3’ (rev)	236 bp	2.05	(38)
***GAPDH***	5’-TGAACGGGAAGCTCACTGG-3’ (fwd) 5’-TCCACCACCCTGTTGCTGTA-3’ (rev)	307 bp	1.86	(39)

### Immunoblotting

MCF-7 BUS cell lysates from siRNA transfections were subjected to electrophoresis on a 10% SDS-polyacrylamide gel, before transfer of proteins to nitrocellulose membranes (AEC Amersham, RSA). The membranes were then probed with primary antibodies specific to ERα (F-10, Santa Cruz Biotechnology, USA), the PR isoforms (NCL-L-PGR-312, Leica Biosystems, Germany) or the loading control, GAPDH (0411, Santa Cruz Biotechnology, USA), followed by incubation with a horseradish peroxidase (HRP)-conjugated secondary antibody (anti-mouse, sc-2005, Santa Cruz Biotechnology, USA). Proteins were visualized using enhanced chemiluminescence (Biorad, RSA) and a MyECL imager and quantified using ImageJ software (version 1.49).

### Chromatin immunoprecipitation and re-ChIP assays

ChIP and re-ChIP assays were conducted as previously described (41), with a few modifications. Briefly, MCF-7 BUS cells were seeded into 10 cm^2^ dishes at a density of 2 x 10^6^ cells in phenol red-free DMEM supplemented with 5% HI-CS-FCS and 100 IU/ml penicillin and 100 μg/ml streptomycin. After 24 hours, the cells were treated with 1 nM P_4_, MPA, NET or DRSP for 2 hours. Following cross-linking of the chromatin and proteins using 1% formaldehyde, cells were harvested in PBS containing protease inhibitors, lysed and sonicated. An aliquot of the sonicated lysate (30 µg) was used as an input control. Approximately 100 µg of chromatin was immunoprecipitated with IgG or antibodies specific to ERα or the PR. Chromatin was collected using pre-blocked protein A/G-PLUS agarose beads. After thorough washing, the DNA-protein complexes were eluted. For ChIP assays, a 1% SDS, 100 mM NaHCO_3_ elution buffer was used, while a 1% SDS, 10 mM dithiothreitol elution buffer containing protease inhibitors was used for re-ChIP assays. For the latter, an aliquot of the supernatant was used as confirmation that the first immunoprecipitation was successful, and the remaining chromatin was re-immunoprecipitated with anti-ERα, anti-PR or anti-IgG antibodies. The cross-linking of all the DNA-protein eluents was then reversed by adding NaCl, followed by incubation overnight at 65°C. Proteinase K (Roche Applied Science, RSA) was added to the samples the following day and incubated at 45°C for 1 hour for protein digestion. The input and immunoprecipitated samples were subsequently purified using the Machery Nagel NucleoSpin^®^ Extract II kit (Separations, RSA) as per the manufacturer’s instructions. Purified DNA samples were analysed by real-time qPCR using the primer sets described in **Table 2**.

**Table 2:** ChIP and re-ChIP primers used for real-time qPCR.

Gene	Primer sequence	Amplicon length	Ref.
***CTSD***	5’-TCCAGACATCCTCTCTGGAA-3’ (fwd) 5’-GGAGCGGAGGGTCCATTC-3’ (rev)	240 bp	(42, 43)
***MYC***	5’-TCTCTGCTGACTCCCCCGGC-3’ (fwd)	71 bp	(24)
	5’-CCGCGGGACCGGACTTCCTA-3’ (rev)		

### Data and statistical analysis

Data analysis, graphical presentations and statistical analysis were performed using GraphPad Prism^®^ version 9 (GraphPad Software). Non-linear regression analysis was used to determine efficacies and potencies. One-way ANOVA analysis of variance with Tukey’s multiple comparison or two-way ANOVA with Bonferroni’s multiple comparison post-tests were used to determine statistical significance of results. The results of at least three independent experiments are shown and the error bars indicate the standard error of the mean (SEM).

## Results

### P_4_, MPA, NET and DRSP increase proliferation and anchorage-independent growth of the estrogen-responsive MCF-7 BUS breast cancer cell line

**Figure 1:**
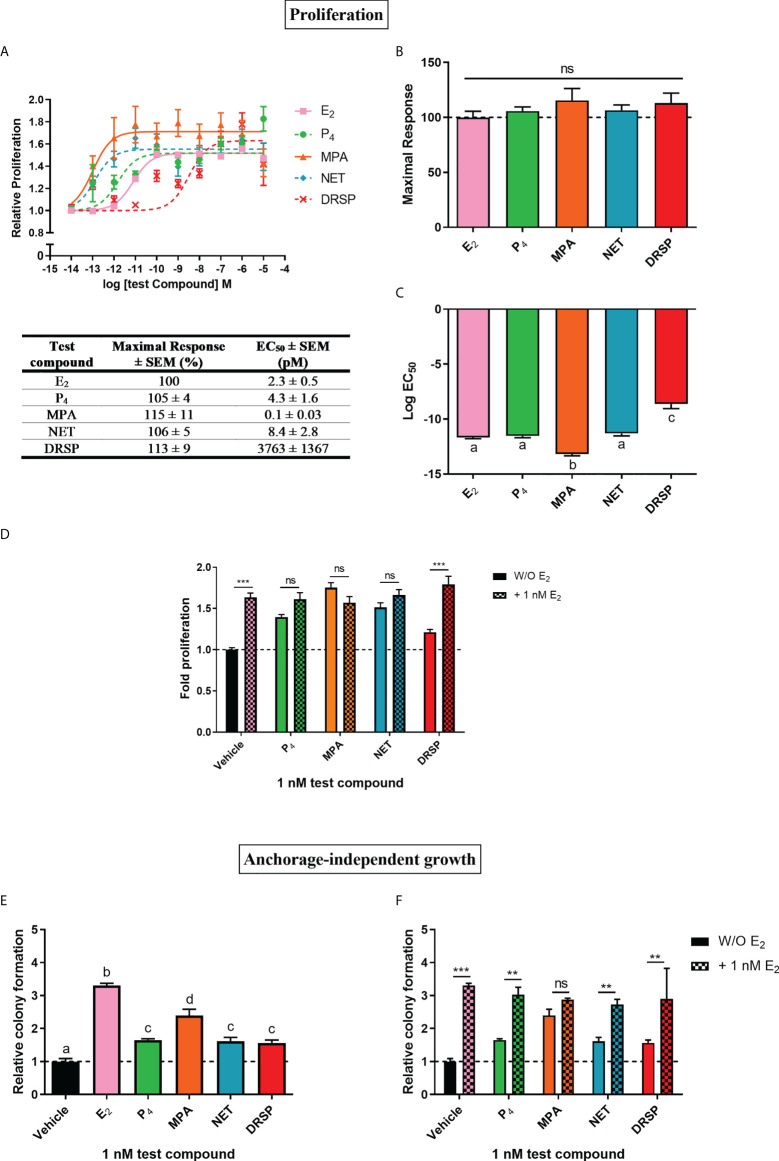
The progestogens display similar efficacies, but not potencies, to each other and E_2_ for increased proliferation of the MCF-7 BUS breast cancer cell line, while enhancing anchorage-independent growth of the MCF-7 BUS cells to differential extents to each other and E_2_. The MCF-7 BUS cell line was incubated for 120 hours with **(A)** increasing concentrations of E_2_ (■), P_4_ (●), MPA (▲), NET (♦) or DRSP (×) or **(D)** 1 nM E_2_ in the absence and presence of 1 nM progestogens. Cell proliferation was quantified using the MTT cell viability assay, and the vehicle response was set as one, with all other responses calculated relative to this. **(B)** Plots of the maximal response and **(C)** log EC_50_ values of the test compounds for proliferation from **(A)** are shown. **(E, F)** MCF-7 BUS cells were treated for 21 days with **(E)** 1 nM E_2_, P_4_, MPA, NET or DRSP, or **(F)** 1 nM progestogen in the presence of 1 nM E_2_. Anchorage-independent growth was quantified using the soft agar assay. The colonies formed were quantified using ImageJ software (Version 1.49). Results are shown as relative colony formation with the response obtained with the vehicle control set as one, and all other responses calculated relative to this. One-way ANOVA with Tukey’s multiple comparison or two-way ANOVA with Bonferroni’s multiple comparison post-tests were used for statistical analysis. Statistically significant differences are indicated by the letters a, b, c or d, where the values that differ significantly from others are assigned a different letter, or ** or *** to indicate p<0.01 or p<0.001. No statistical significance (p>0.05) is indicated by ns. One-way ANOVA with a Dunnett’s multiple comparison post-test was performed to compare responses of estrogen-progestogen combinations relative to E_2_ alone, and no statistical differences were obtained between the E_2_ response and E_2_ in combination with any progestogen.

Progestins often elicit biological effects that are distinct from each other and natural P_4_ [reviewed in (2, 44, 45)]. Indeed, it is known that some progestins have been linked to increased breast cancer risk, while oral micronized P_4_ has not [reviewed in (14, 17)]. Here, we directly compared the effects of the older first-generation progestins MPA and NET, as well as the newer fourth-generation progestin DRSP, relative to each other and natural P_4_ on cell proliferation (**Figures 1A–C**) and anchorage-independent growth (**Figure 1E**) in the MCF-7 BUS breast cancer cell line. Although NET-A is administered in MHT, we used its active metabolite (46) in our study, as it has previously been shown that NET-A and NET elicit similar effects to each other (47). MPA and NET were included in this study as they have both been linked to increased breast cancer incidence [reviewed in (14)], and are known to differentially activate steroid receptors such as the glucocorticoid receptor (GR) (47, 48) and ERα (5). DRSP was included as we have previously shown that it elicits effects similar to P_4_, but dissimilar to MPA and NET-A (5, 49). For proliferation assays, the cells were incubated with increasing concentrations of E_2_, P_4_, MPA, NET and DRSP (**Figure 1A**) and proliferation quantified using the MTT cell viability assay. Since the MCF-7 BUS breast cancer cell line is estrogen-sensitive and highly proliferative in response to E_2_ treatment (50), we included treatment with E_2_ alone as a positive control. Surprisingly, the selected progestins and P_4_ had similar efficacies to each other and E_2_ for proliferation of the MCF-7 BUS cells (**Figures 1A, B**). While P_4_ and NET were equipotent to each other and E_2_, MPA was approximately 20-fold more potent than E_2_, and DRSP approximately 1600-fold less potent (**Figures 1A, C**). The maximal responses (efficacies) and EC_50_ values (potencies) for proliferation are summarized in the table in **Figure 1A**. Notably, proliferation in response to 1 nM DRSP was significantly lower than that observed for the other progestins and P_4_ (**Supplementary Figure 1A**).

We also investigated progestogen effects in the presence of E_2_. These experiments were crucial as progestogens are used in combination with an estrogen in MHT (14), and it has been argued that experiments with P_4_ and the PR should include conditions where the individual and combinatorial effects of estrogen and the progestogen are investigated, as physiologically, women are always exposed to both P_4_ and some level of endogenous estrogens (51). We examined the effects in the presence of equimolar concentrations of E_2_ and the progestogens, as well as in the presence of 100x more progestogen than E_2_, which is representative of the ratios in which these hormones are used in MHT [reviewed in (45)]. Our results show that the progestogens did not inhibit E_2_-induced proliferation, while the addition of E_2_ did not modulate the proliferative effects of either 1 or 100 nM P_4_, MPA and NET (**Figure 1D**; **Supplementary Figure 1B**). In contrast, the proliferative effects of 1 nM, but not 100 nM, DRSP, was significantly different in the presence of E_2_ (**Figure 1D**), likely due to the effects of E_2_ only.

Since the ability of tumor cells to survive and grow anchorage-independently is essential for metastasis, the soft agar assay was used to quantify the number of colonies formed in response to 1 nM progestogens in the absence (**Figure 1E**) and presence of 1 nM E_2_ (**Figure 1F**). Results show that all progestogens increased colony number, albeit to a lesser extent than E_2_ (**Figure 1E**). In line with the proliferation results (**Supplementary Figure 1A**), the number of colonies formed in response to 1 nM MPA was significantly greater than that of 1 nM P_4_, NET or DRSP. Notably, no differences in the number of colonies were observed for the test compounds at 100 nM (**Supplementary Figure 1C**). While the number of colonies were similar for MPA in the absence and presence of E_2_, a significant increase in colonies, like the effects of E_2_ only, were observed when P_4_, NET and DRSP were combined with E_2_ (**Figure 1F**). As observed for proliferation, 1 nM E_2_ did not modulate the effects of 100 nM of the progestogens on anchorage-independent growth (**Supplementary Figure 1D**), neither did 1 or 100 nM progestogens influence the E_2_-induced anchorage-independent growth of the MCF-7 BUS cells (**Figure 1F** and **Supplementary Figure 1C**).

### The regulation of the ER-regulated CTSD gene by MPA and NET requires both PR and ERα

**Figure 2:**
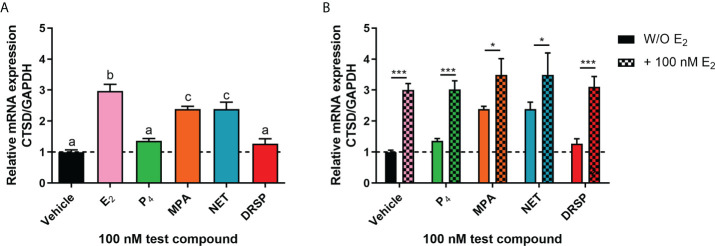
The progestogens differentially regulate *CTSD* mRNA expression which is modulated by E_2_. The MCF-7 BUS cell line was treated with **(A)** 100 nM E_2_, P_4_, MPA, NET or DRSP or **(B)** equimolar concentrations of progestogen and E_2_ for 24 hours. Total RNA was isolated, reverse transcribed and real-time qPCR conducted to determine the relative expression of *CTSD* mRNA levels relative to that of *GAPDH* (reference gene). The vehicle control was set as one and the relative mRNA expression of *CTSD* in the treated samples set relative to this. One-way ANOVA with Tukey’s multiple comparison or two-way ANOVA with Bonferroni’s multiple comparison post-tests were used for statistical analysis. Statistically significant differences are indicated by the letters a, b or c, where the values that differ significantly from others are assigned a different letter, or * or *** to indicate p<0.05 or p<0.001. One-way ANOVA with a Dunnett’s multiple comparison post-test was performed to compare responses of estrogen-progestogen combinations relative to E_2_ alone, and no statistical differences were obtained between the E_2_ response and E_2_ in combination with any progestogen.

**Figure 3:**
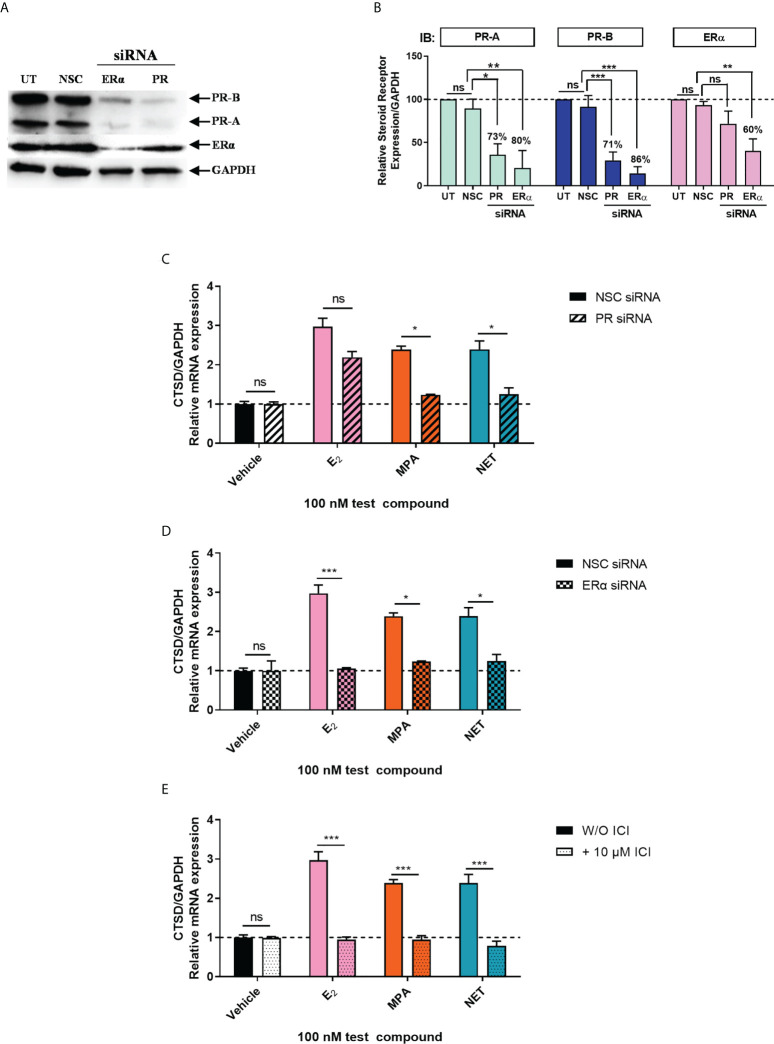
Both the PR and ERα are required for the upregulation of the ER-regulated gene *CTSD* by MPA and NET. The MCF-7 BUS cell line transfected with **(A-C)** 10 nM NSC or PR-A/B siRNA or **(A, B, D)** 25 nM NSC or ERα siRNA were treated with 100 nM E_2_, MPA or NET for 24 hours. **(A)** For verification of PR-A/B or ERα knockdown, total protein from the MCF-7 BUS cells transfected as described above was harvested, and western blotting performed using antibodies specific for ERα, PR-A/B and GAPDH. A representative blot is shown and **(B)** PR-A, PR-B and ERα expression levels were quantified relative to the GAPDH loading control using ImageJ software (Version 1.49). Western blots of three independent experiments were quantified to determine the percentage protein knocked down. **(E)** MCF-7 BUS cells were treated with 100 nM E_2_, MPA or NET in the absence and presence of 10 µM ICI for 24 hours. **(C–E)** Total RNA was isolated, reverse transcribed and real-time qPCR was conducted to determine the relative expression of *CTSD* mRNA levels relative to *GAPDH* (reference gene). The vehicle control of each condition was set as one and the relative mRNA expression in the treated samples set relative to this. Two-way ANOVA with Bonferroni’s multiple comparison post-test was used for statistical analysis. Statistically significant differences are indicated by *, ** or *** to indicate p<0.05, p<0.01 or p<0.001. No statistical significance (p>0.05) is indicated by ns.

Considering that biological phenotypes of cancer, such as cell proliferation and anchorage-independent growth, are mirrored by changes in gene expression (52), we next investigated whether the selected progestogens could modulate the expression of the known ER-regulated *CTSD* gene. Results show that P_4_ and DRSP had no effect on *CTSD* mRNA expression, while both MPA and NET significantly increased the expression, albeit to a lesser extent than E_2_ (**Figure 2A**). The increase in *CTSD* gene expression observed when E_2_ was added to P_4_ and DRSP was most likely because of E_2_. While E_2_-induced *CTSD* mRNA expression was not inhibited by equimolar concentrations of the progestogens, E_2_ enhanced the MPA- and NET-induced upregulation of *CTSD* mRNA expression (**Figure 2B**). Knowing that both MPA and NET can bind to the PR, whilst NET-A, but not MPA, can bind to ERα (5), we next silenced the expression of the PR isoforms and ERα to assess their roles in the MPA and NET-mediated regulation of the *CTSD* gene. Western blotting (**Figures 3A, B**) confirmed that transfection of the MCF-7 BUS cell line with PR-A/B siRNA resulted in a 73% and 71% knockdown of PR-A and PR-B respectively, while ERα siRNA resulted in a 60% decrease in ERα expression. Both PR and ERα knockdown abrogated MPA- and NET-induced *CTSD* mRNA expression (**Figures 3C, D**). As expected, the E_2_-induced increase in *CTSD* mRNA expression was inhibited when ERα was silenced (**Figure 3D**). We also showed that silencing of ERα resulted in 80% knockdown of PR-A and 86% knockdown of PR-B (**Figures 3A, B**), which raised the question as to whether both the PR and ER, or only the PR, are required. To exclude the latter, we confirmed that the ER is indeed required by showing that the effects of MPA and NET on *CTSD* gene expression are abrogated in the presence of the ER antagonist, ICI-182,780 (**Figure 3E**), which does not decrease PR levels in MCF-7 cells (53). Consistent with our findings that both the PR and ER are required for regulation of *CTSD* gene expression by MPA and NET, both receptors were required for *CTSD* transcription by the progestin, promegestone (R5020), in MCF-7 cells (53).

### The PR and ERα are co-recruited to both the CTSD and MYC promoters

**Figure 4:**
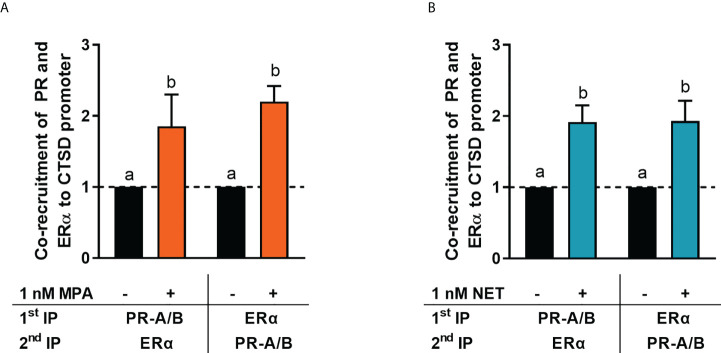
ERα and the PR are co-recruited to the *CTSD* promoter in response to MPA and NET. The MCF-7 BUS cell line was incubated with 1 nM **(A)** MPA or **(B)** NET for 2 hours followed by re-ChIP assays. Cell lysates were subjected to immunoprecipitation (IP) with an anti-IgG antibody (negative control) or a PR-A/PR-B-specific antibody followed by an ERα-specific antibody, and vice versa, prior to real-time qPCR analysis of the resulting immunoprecipitated DNA fragments and input controls. Data shown was normalized to input and IgG controls and expressed as the fold response relative to the vehicle control set as one. One-way ANOVA with Tukey’s multiple comparison post-test was used for statistical analysis. Statistically significant differences are indicated by the letters a or b, where the values that differ significantly from others are assigned a different letter.

**Figure 5:**
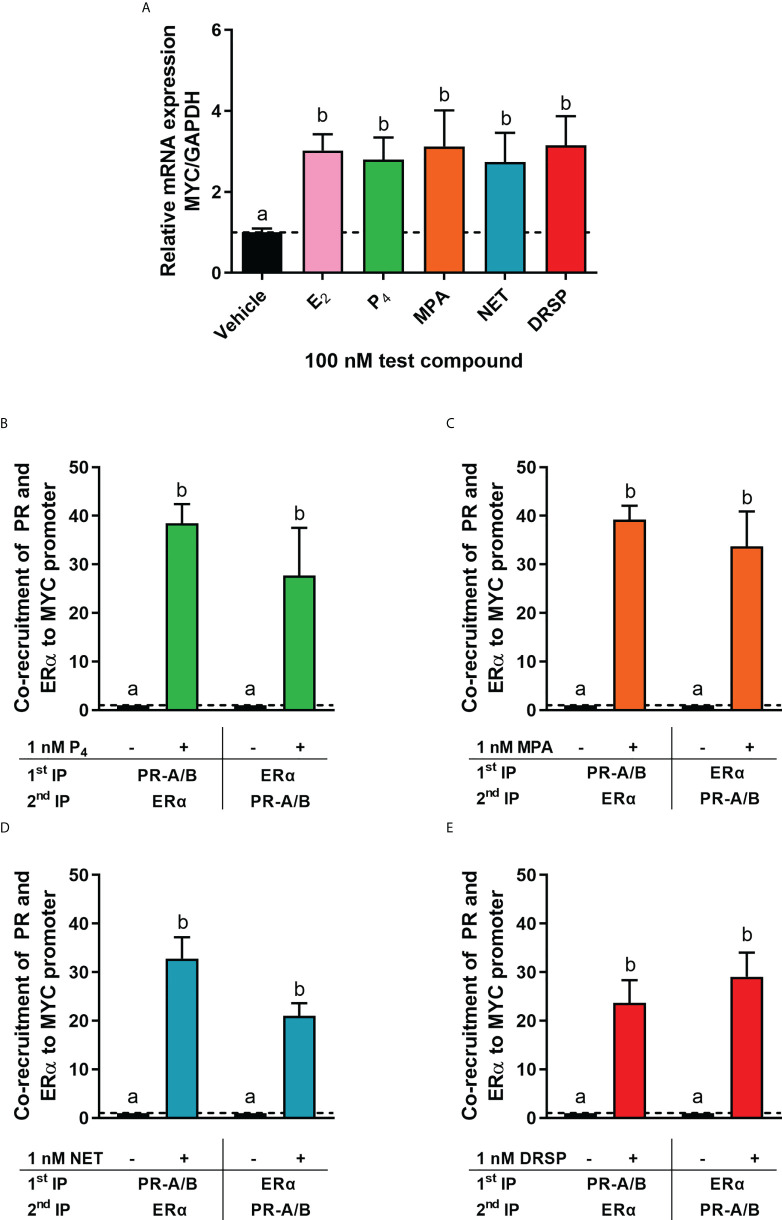
The progestogens upregulate *MYC* mRNA expression to the same extent as each other and E_2_ and induce co-localization of the PR and ERα on the *MYC* promoter. **(A)** The MCF-7 BUS cell line was treated with 100 nM E_2_, P_4_, MPA, NET or DRSP for 24 hours. Total RNA was isolated, reverse transcribed and real-time qPCR conducted to determine the relative expression of *MYC* mRNA levels relative to that of *GAPDH* (reference gene). The vehicle control was set as one and the relative mRNA expression of *MYC* in the treated samples set relative to this. **(B–E)** The MCF-7 BUS cell line was incubated with 1 nM P_4_, MPA, NET or DRSP for 2 hours followed by re-ChIP assays. Cell lysates were subjected to immunoprecipitation (IP) with an anti-IgG antibody (negative control), or a PR-A/PR-B-specific antibody followed by an ERα-specific antibody, and vice versa, prior to real-time qPCR analysis of the resulting immunoprecipitated DNA fragments and input controls. Data shown was normalized to input and IgG controls and expressed as the fold response relative to the vehicle control set as one. One-way ANOVA with Tukey’s multiple comparison post-test was used for statistical analysis. Statistically significant differences are indicated by the letters a or b, where the values that differ significantly from others are assigned a different letter.

It is known that the PR and ERα can occur in a complex both in the absence and presence of ligand (24, 28). Thus, we next investigated whether MPA or NET treatment would cause both the PR and ERα to be recruited to the promoter of the endogenous *CTSD* gene, as only these two progestins increased *CTSD* gene expression. MCF-7 BUS cells were incubated for 2 hours with MPA, or NET, and then subjected to immunoprecipitation with an anti-IgG antibody (negative control) or a PR-A/B-specific antibody followed by an ERα-specific antibody, and vice versa. Results show that the PR and ERα are co-localized on the endogenous *CTSD* promoter in the presence of MPA (**Figure 4A**) and NET (**Figure 4B**). As co-localization of these receptors has previously been shown on PR binding sites in the promoter of the progestogen-responsive proto-oncogene *MYC* in response to MPA in the T47D breast cancer cell line (24), we first investigated the regulation of *MYC* mRNA expression in response to our panel of progestogens in the MCF-7 BUS cell line, and subsequently whether these ligands would induce PR and ERα co-localization on the *MYC* promoter. As shown in **Figure 5A**, P_4_, MPA, NET and DRSP increased *MYC* mRNA expression to a similar extent as each other and E_2,_ and these effects were not modulated in the presence of E_2_ or vice versa (**Supplementary Figure 2**). We show that MPA treatment induced PR and ERα co-localization on the *MYC* promoter in MCF-7 BUS cells (**Figure 5C**), consistent with previous findings in T47D cells (24). This co-localization was not unique to MPA but was also observed for P_4_ (**Figure 5B**), NET (**Figure 5D**) and DRSP (**Figure 5E**). Notably, 1 nM E_2_ did not lead to significant co-recruitment of the PR and ERα to the *MYC* promoter (**Supplementary Figure 3**).

## Discussion

Considering that breast cancer is the leading cause of mortality in women in developed countries (54–56), establishing the mechanism underlying the increased breast cancer risk associated with estrogen-progestin MHT is a global priority. Since estrogen-progestin MHT is associated with higher breast cancer risk than estrogen-only MHT (8, 12), it is clear that the progestin component is the bigger culprit. Molecular studies are thus urgently required to directly compare the effects of progestins relative to each other and P_4_ in breast cancer. In this study, the frequently used MTT assay was employed as a measure of proliferation of the MCF-7 BUS breast cancer cell line. Although MCF-7 cells are widely used as a model to evaluate hormonal effects on breast cancer cell growth, we used the MCF-7 BUS sub-clone as it has been shown to be the most proliferative in response to E_2_ when compared to other MCF-7 cells (50). We showed that the older first-generation progestins MPA and NET, the newer fourth-generation progestin DRSP, and natural P_4_ all increase proliferation of the estrogen-responsive MCF-7 BUS breast cancer cell line with similar efficacies, but not potencies (**Figures 1A–C**). DRSP was 875-fold less potent than P_4_, 37 630-fold less potent than MPA and 448-fold less potent than NET. Not only was MPA the most potent progestogen in terms of proliferation, but it was also 20-fold more potent than E_2_. Given that E_2_ is a known mitogen in promoting cellular proliferation in the breast, our results may explain, at least in part, the increased breast cancer incidence observed with the combination of E_2_ and MPA in MHT. An important validation of our results would be to correlate the absorbance determined by MTT to the cell number in future studies, as it is plausible that the true proliferation may be obscured by the progestogens influencing the metabolic activity of the cells. In agreement with our findings, however, previous studies have shown increased proliferation with both MPA (24, 57) and NET (57, 58). However, at least one study has shown anti-proliferative effects for both MPA and NET (59). In contrast to the older generation progestins, studies investigating the effects of DRSP on breast cancer hallmarks are scarce. To our knowledge, only one study has shown that DRSP increases the growth of human breast epithelial xenografts (60). While some clinical studies have reported that P_4_ is not associated with an increased risk of breast cancer, others have indicated an increase [reviewed in (45)]. In line with these contradictory findings, results from some experimental studies have shown increased proliferation with P_4_ (61–63), while others have shown anti-proliferative effects (64, 65). The above-mentioned studies reporting differential effects on proliferation by the progestogens in different experimental cell models, highlight the importance of comparing progestogen activities in parallel in the same model system. While little is known about the effects of P_4_ and these progestins on metastasis, we showed that 1 nM MPA increased the anchorage-independent growth of the MCF-7 BUS cell line to a greater extent than P_4_, NET and DRSP (**Figure 1E**), suggesting that MPA has a greater metastatic potential than the other progestogens. Consistent with this result, a previous study has shown that MPA increased migration and invasion of the T47D breast cancer cell line to a greater extent than P_4_ and DRSP (66).

To further understand the mechanism of these progestogens in breast cancer, we investigated whether P_4_, MPA, NET and DRSP could regulate the expression of the ER-regulated *CTSD* gene. *CTSD* has been linked to breast cancer metastasis, invasion, relapse and short disease survival (67). Our results show that the progestogens differentially regulated the mRNA expression of *CTSD*, with P_4_ and DRSP having no effect, whereas MPA and NET upregulated its expression (**Figure 2**). Preliminary studies in the T47D cell line showed similar findings whereby MPA and NET upregulated *CTSD* mRNA expression, but DRSP did not (**Supplementary Figure 4**). Further studies in the T47D cell line or another ER/PR positive breast cancer cell line such as ZR-75-1 would be valuable to strengthen our findings. We subsequently showed that the effects of MPA and NET were abrogated when the expression of PR and ERα was silenced, suggesting that both the PR and ERα are required for MPA- and NET-induced upregulation of *CTSD* gene expression (**Figures 3C, D**). While it has been indicated that the PR can decrease the expression of ER target genes involved in cell cycle progression (28), our findings on the ER-regulated *CTSD* gene suggest that the PR does not lead to a reduction in the expression of all ER target genes. Considering that we have previously shown that NET-A, but not MPA, can bind to ERα (5), our results suggest that at least the MPA-induced mRNA expression does not occur *via* a mechanism requiring binding to the ER, but rather suggests an indirect role for the ER. Using sequential ChIP assays, we are the first to show that MPA- and NET-induced upregulation of the ER-regulated *CTSD* gene, *via* a mechanism requiring the recruitment of both the PR and ERα to the *CTSD* gene promoter (**Figure 4**). It is known that the *CTSD* promoter contains different *cis*-elements (67, 68) to which steroid receptors can bind (53, 69–71). For example, ERα can increase transcription by binding to the estrogen response element (ERE) (72, 73) or by tethering to activator protein (AP)-1 bound to an AP-1 element in the *CTSD* promoter (69, 70). Specific protein (Sp)-1 binding sites are also found in the *CTSD* promoter, and it has previously been shown that the PR increases the expression of the *PR* (53) and *p21* (71) genes *via* an indirect interaction with these sites. Furthermore, the PR has previously been shown to interact with an ERE/Sp1 site in the *PR* promoter (53). Although we did not delineate the precise mechanism whereby the PR/ERα complex mediates the regulation of the ER target gene by MPA and NET, it may be that the PR/ERα complex occupies the ERE/Sp1 site in the *CTSD* promoter. In contrast to our observations on the *CTSD* promoter, we show that the PR and ERα are co-recruited to the PR-regulated *MYC* gene promoter in response to all the progestogens, resulting in the upregulation of *MYC* mRNA expression (**Figure 5**). These results suggest that the previously reported PR and ERα co-recruitment to the *MYC* promoter in the T47D breast cancer cell line in response to MPA treatment (24) is neither cell line- nor progestogen-specific. Considering that the expression of *MYC* is often upregulated in breast cancer, and that it plays a role in promoting proliferation (74, 75), these results suggest that the progestogens evaluated in this study all promote breast cancer cell proliferation, albeit to different extents, *via* a mechanism requiring an association of the PR and ERα on the *MYC* promoter. Although activation of the PR in the presence of an estrogen-activated ER complex has been associated with a more favorable outcome (28, 31), one cannot ignore the fact that the PR/ER complex is recruited to the PR-regulated *MYC* oncogene, and that PR-regulated genes have previously been linked to increased tumor progression (19). It is therefore critical that the manner in which PR and ER pathways cooperate to modulate the expression of ER and PR-regulated genes in response to different progestins is understood. Future studies should include investigations into similar mechanisms on other ER and PR target genes such as Trefoil Factor 1 (TFF1), cyclin D1, tissue factor (CD142), or Serum/Glucocorticoid Regulated Kinase 1 (SGK).

We also investigated the effects of a combination of progestogen and E_2_ on cell proliferation, anchorage-independent growth and gene expression, as it has been argued that the estrogenic status directly influences whether progestogens are anti- or pro-proliferative (17). While it has been reported that the progestogens P_4_ (76, 77), MPA (76–79), NET (77–79) and DRSP (76) decrease E_2_-induced proliferation of the ER/PR positive HCC1500, T47D or MCF-7 breast cancer cell lines, others have shown that P_4_ (63, 80), MPA and NET (80) increase E_2_-induced cell growth of BT474 or T47D xenograft tumors in mice, and that P_4_ increases E_2_-induced anchorage-independent growth of MCF-7 cells (62). In contrast, we showed that none of the progestogens influenced E_2_-induced cell proliferation or anchorage-independent growth of the MCF-7 BUS cells (**Figure 1**). Similarly, none of the progestogens modulated the E_2_-induced upregulation of the *MYC* (**Supplementary Figure 2**) gene. Although the progestin effects on *MYC* gene expression was not modulated by E_2_, the combination of E_2_ and DRSP on proliferation, as well as E_2_ and P_4_, NET or DRSP on anchorage-independent growth, resembled the response of E_2_ alone. Likewise, none of the progestogens modulated E_2_-induced upregulation of the *CTSD* gene (**Figure 2B**), whereas the effects of the combination of E_2_ with each progestogen resembled the E_2_ alone response. Another key issue recently raised is the physiologically relevant concentration of progestogens to use in experiments (51, 81). Indeed, differential effects observed between studies investigating progestogen effects may be ascribed not only to different experimental models used, but also differences in concentrations of ligand used. While it has been recommended that only concentrations between 1 and 10 nM can be considered physiologically relevant (51), it cannot be excluded that higher concentrations of progestin may be found in breast tissue as circulating progestin levels do not necessarily reflect intramammary tissue levels. To our knowledge, the levels of MPA, NET and DRSP in breast tissue have not been determined and should be urgently addressed to accurately correlate these levels with biological responses in breast cancer biology.

Several studies have investigated crosstalk between the PR and ERα in breast cancer etiology (24, 25, 28, 29, 31, 32). Some have shown that the interaction between liganded ERα and the unliganded (25) or MPA-activated PR-B (24) results in the upregulation of ER- or PR-regulated target genes, as well as breast cancer cell proliferation. Genome-wide studies however showed that when the PR is bound to the PR agonists, P_4_ or R5020, the PR modulates the chromatin localization of the estrogen activated ERα (28, 29), resulting in a gene expression profile similar to that of PR alone, and one that is associated with decreased proliferation and an improved clinical outcome (28). These former studies resulted in a renewed interest in using PR agonists (P_4_ or progestins) in ER-positive breast cancer therapy, by combining these ligands with ER-targeted ligands (17). Consistent with the idea that PR agonists are associated with a good prognosis, it has also been shown that P_4_ or MPA-activated PR inhibits E_2_-induced tumor growth and the expression of ER-regulated genes in breast cancer patient-derived xenografts (31). However, suitability of individual progestins would need to be assessed considering that some progestins are associated with increased breast cancer risk (14). The subsequent Singhal study showed that the role of the PR in ER-positive breast cancer is not straightforward, as modulation of estrogen signaling by the PR resulted in either pro- or anti-tumorigenic effects depending on the PR isoform involved, and whether the PR isoform was bound to an agonist or antagonist (32). Gene expression analysis from patient cohorts predicted that unlike the regulation of genes by PR agonists, regulation by PR antagonists is associated with a better survival outcome. The findings by Singhal and co-workers emphasized the need for more mechanistic studies to aid in a complete understanding of the complexity of PR and ERα crosstalk in breast cancer, in the drive to develop PR/ER co-therapies for ER-positive breast cancer. Our findings that the PR and ERα are co-recruited to the PR target gene in response to all progestogens, while recruitment to the ER target gene is progestin-specific, underscores the importance of investigating the clinical relevance of the interaction between ERα and the PR in response to multiple progestins in parallel in both normal and malignant breast tissue. This is especially important considering the reservations about using progestins in breast cancer therapy given their association with increased breast cancer incidence. It is important to note that these reservations are based on observations of clinical studies linking only eight progestins to increased incidence of breast cancer (14), whereas there are a large number of clinically available progestins yet to be evaluated in terms of breast cancer risk. Our results indicating differential progestin effects highlight the importance of directly comparing the effects of individual progestins, rather than grouping them as a class. Finally, further *ex vivo* and *in vivo* experiments will be critical to appreciate the physiological implications of our findings, and to understand the role of individual progestins in processes such metastasis.

## Data availability statement

The original contributions presented in the study are included in the article/**Supplementary Material**. Further inquiries can be directed to the corresponding author.

## Author contributions

MP, RL-dT and DA made substantial contributions to conception and design of the study, as well as intellectual input into the manuscript. MP, RL-dT, HJ and MS were involved in data acquisition. MP, RL-dT and DA were involved in data analysis. MP and RL-dT drafted the manuscript, and all authors were involved in reading and editing of the manuscript. All authors contributed to the article and approved the submitted version.

## Funding

This work was funded by the National Research Foundation (NRF) in South Africa (grant number 99114) and the Medical Research Council of South Africa. The funders had no role in study design, data collection and analysis, interpretation of data, decision to publish, or preparation of the manuscript. Any opinion, findings and conclusions or recommendations expressed in this material are those of the authors, and therefore the NRF does not accept any liability in regard thereto.

## Acknowledgments

We thank Carmen Langeveldt for maintaining the MCF-7 BUS cell line.

## Conflict of interest

The authors declare that the research was conducted in the absence of any commercial or financial relationships that could be construed as a potential conflict of interest.

## Publisher’s note

All claims expressed in this article are solely those of the authors and do not necessarily represent those of their affiliated organizations, or those of the publisher, the editors and the reviewers. Any product that may be evaluated in this article, or claim that may be made by its manufacturer, is not guaranteed or endorsed by the publisher.
