# Supplementary material for: Upregulation of an estrogen receptor-regulated gene by first generation progestins requires both the progesterone receptor and estrogen receptor alpha
Source: Front Endocrinol (Lausanne). 2022 Sep 15;13:959396. doi: 10.3389/fendo.2022.959396 (PMC9519895; doi:10.3389/fendo.2022.959396)
Supplement: Supplementary file 1 [file DataSheet_1.docx]

Supplementary Material

## Supplementary Figures

**Proliferation**

**A**

**B**


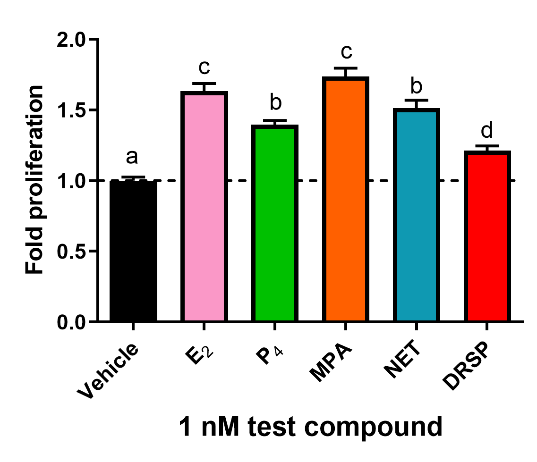

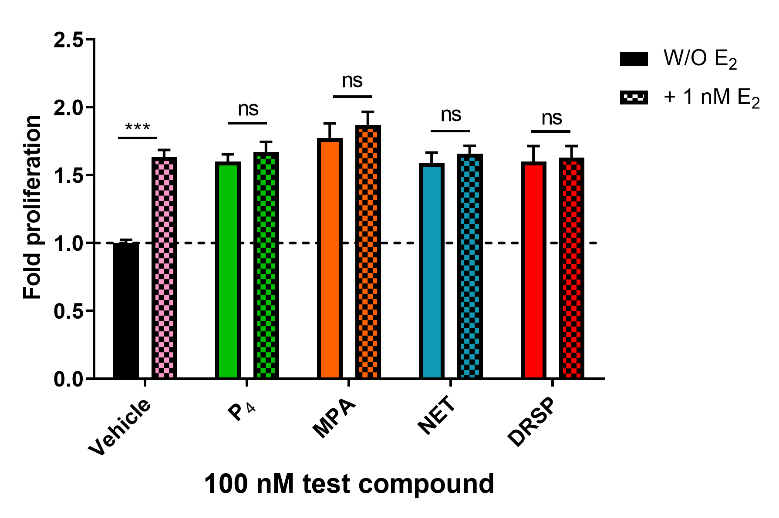


**Anchorage-independent growth**

**D**

**C**


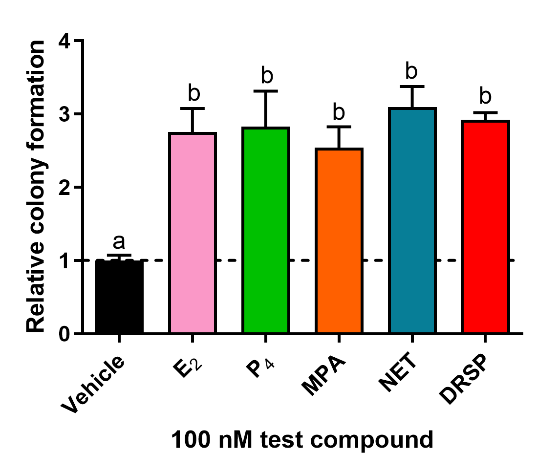

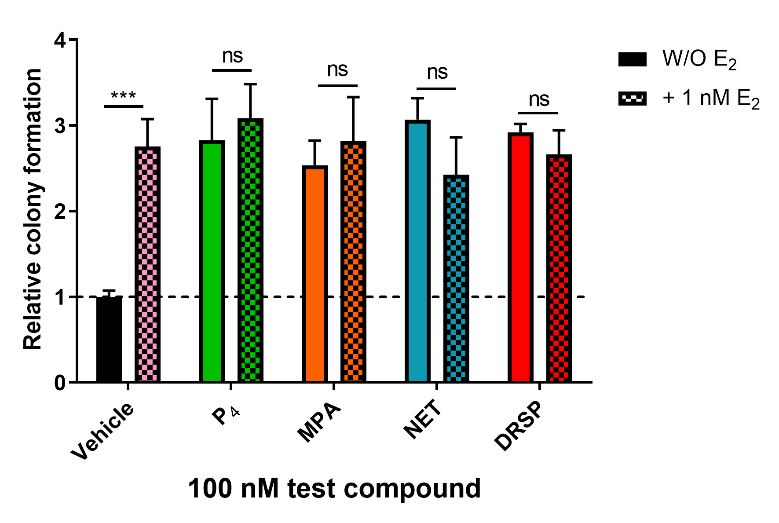


**Supplementary Figure 1. Progestogens do not inhibit E_2_-induced proliferation and anchorage-independent growth of the MCF-7 BUS breast cancer cell line, and progestogen effects are not influenced by E_2_.** The MCF-7 BUS cell line was incubated for 120 hours with **(A)** 1 nM or **(B)** 100 nM P_4_, MPA, NET or DRSP, in the absence and presence of 1 nM E_2,_ or E_2_ only. Cell proliferation was quantified using the MTT cell viability assay, with the vehicle response set as one and all other responses calculated relative to this. **(C and D)** MCF-7 BUS cells were incubated for 21 days with **(C)** 100 nM E_2_, P_4_, MPA, NET or DRSP, or **(D)** 100 nM progestogen in the absence and presence of 1 nM E_2_. Anchorage-independent growth was quantified using the soft agar assay and the colonies formed quantified using ImageJ software (Version 1.49). Results are shown as relative colony formation with the vehicle response set as one, and all other responses calculated relative to this. One-way ANOVA with Tukey’s multiple comparison or two-way ANOVA with Bonferroni’s multiple comparison post-tests were performed for statistical analysis. Statistically significant differences are indicated by the letters a, b, c or d, where the values that differ significantly from others are assigned a different letter, or by *** to indicate p<0.001. No statistical significance (p>0.05) is indicated by ns.


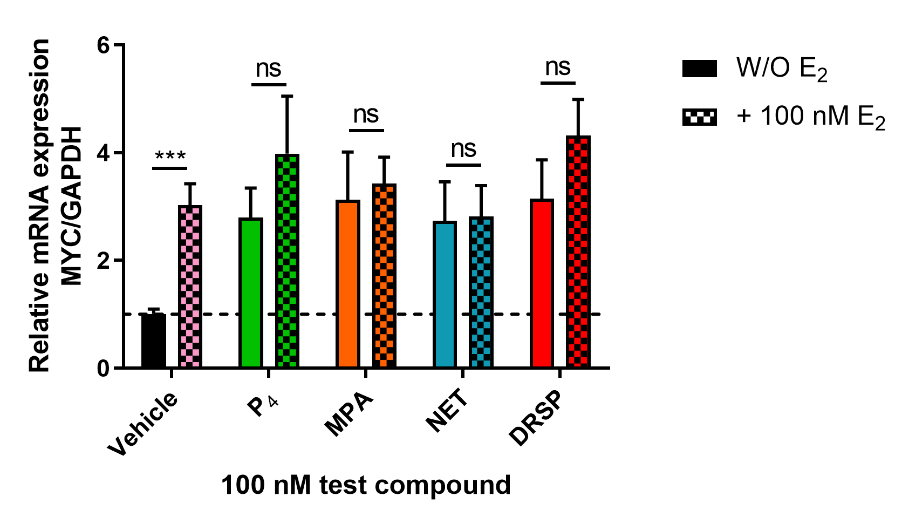


**Supplementary Figure 2. E_2_ does not modulate the effects of the progestogens on MYC mRNA expression or vice versa.** The MCF-7 BUS cell line was treated with 100 nM P_4_, MPA, NET or DRSP in the absence or presence of 100 nM E_2_ for 24 hours. Total RNA was isolated, reverse transcribed and real-time qPCR conducted to determine the relative expression of *MYC* mRNA levels relative to that of *GAPDH* (reference gene). The vehicle control was set as one and the relative mRNA expression of *MYC* in the treated samples set relative to this. Two-way ANOVA with Bonferroni’s multiple comparison post-test was used for statistical analysis. Statistically significant differences are indicated by *** to indicate p<0.001. No statistical significance (p>0.05) is indicated by ns.

**
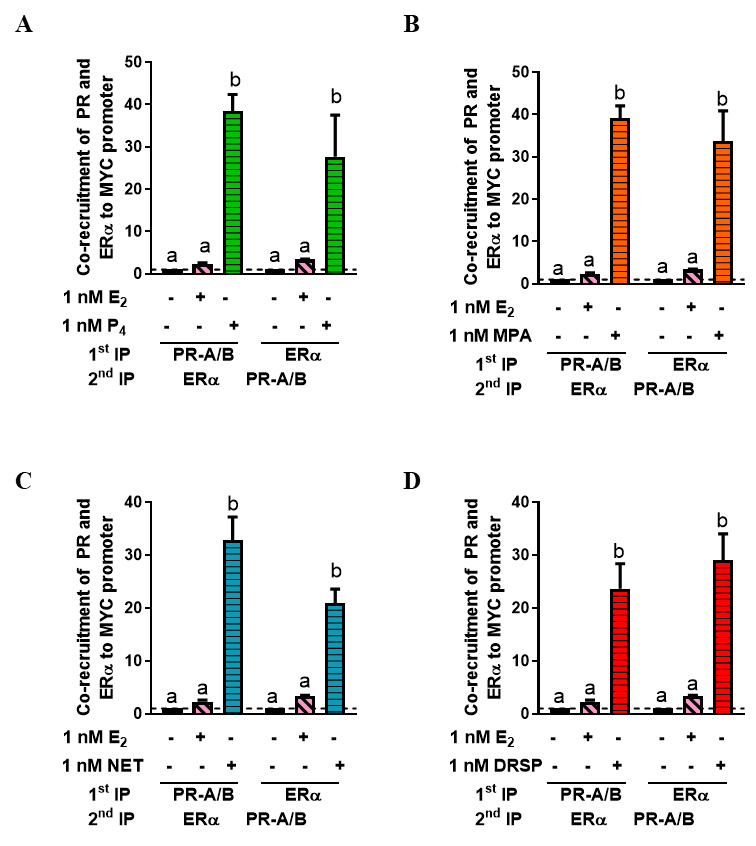
**

**Supplementary Figure 3. E_2_ does not lead to significant co-recruitment of the PR and ERα to the MYC promoter.** The MCF-7 BUS cell line was incubated with **(A-D)** 1 nM E_2_ or **(A)** P_4_ or **(B)** MPA or **(C)** NET or **(D)** DRSP for 2 hours followed by re-ChIP assays. Cell lysates were subjected to immunoprecipitation (IP) with an anti-IgG antibody (negative control), or a PR-A/PR-B-specific antibody followed by an ERα-specific antibody, and vice versa, prior to real-time qPCR analysis of the resulting immunoprecipitated DNA fragments and input controls. Data shown was normalized to input and IgG controls and expressed as the fold response relative to the vehicle control set as one. One-way ANOVA with Tukey’s multiple comparison post-test was used for statistical analysis. Statistically significant differences are indicated by the letters a or b.


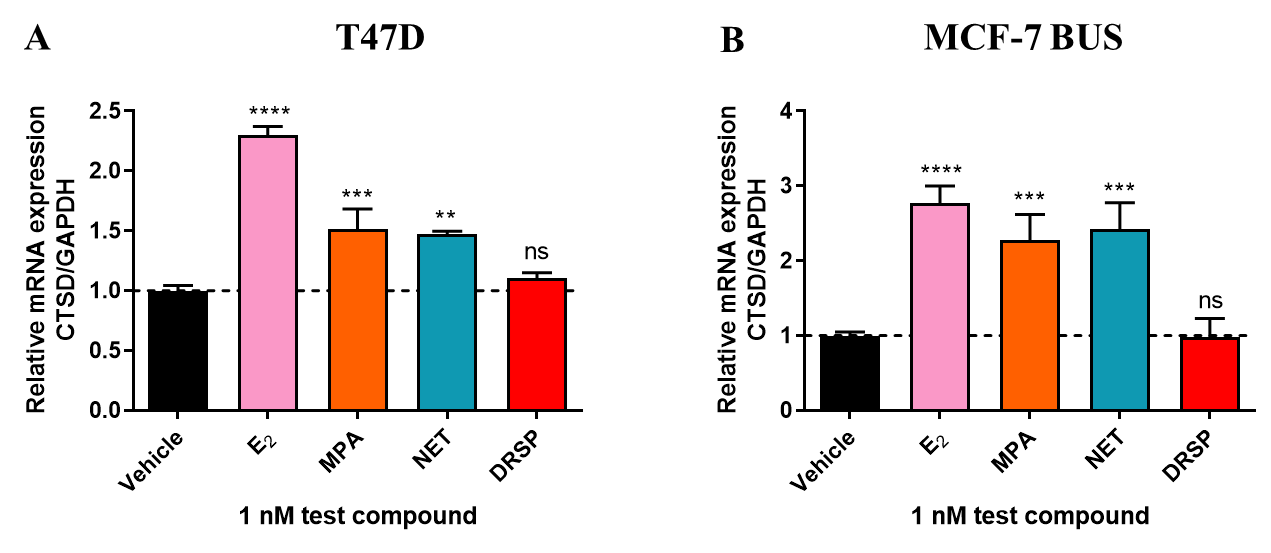


**Supplementary Figure 4. E_2,_ MPA and NET, but not DRSP, upregulate *CTSD* mRNA expression in both MCF7-BUS and T47D breast cancer cell lines.** The **(A)** T47D and **(B)** MCF-7 BUS breast cancer cell lines were treated with 1 nM E_2_, MPA, NET or DRSP for 24 hours. Total RNA was isolated, reverse transcribed and real-time qPCR conducted to determine the relative expression of *CTSD* mRNA levels relative to that of *GAPDH* (reference gene). The vehicle control was set as one and the relative mRNA expression of *CTSD* in the treated samples set relative to this. One-way ANOVA with Dunnett’s post-test (comparing all responses relative to the vehicle) was used for statistical analysis. Statistically significant differences are indicated by **, *** or **** to indicate p<0.01, p<0.001 or p<0.0001, respectively. No statistical significance (p>0.05) is indicated by ns.
